# Circadian and Sex-Specific Responses to *Alternaria alternata* in Allergic Airway Inflammation

**DOI:** 10.64898/2026.09.30.755795

**Published:** 2026-10-07

**Authors:** Santhosh Kumar Duraisamy, Joanna Mainzer, Chandrashekar Prasad, Steven Haworth, Mario Castro, Isaac Kirubakaran Sundar

## Abstract

Asthma severity and immune responses are regulated by circadian rhythms, yet the role of circadian timing in fungal allergen-induced airway inflammation remains poorly defined. *Alternaria alternata* , a clinically relevant fungal allergen, is strongly associated with severe asthma exacerbations and persistent airway dysfunction. Using a preclinical mouse model, we investigated whether pulmonary responses to *Alternaria* exposure are influenced by time of day and sex. We demonstrate that *Alternaria* induces allergic airway inflammation, airway hyperresponsiveness (AHR), and epithelial barrier disruption in a circadian- and sex-dependent manner. Female mice exhibited enhanced eosinophilic, neutrophilic, interstitial macrophage, and dendritic cell infiltration predominantly at Zeitgeber Time 0 (ZT0), whereas males showed stronger inflammatory responses at ZT12. *Alternaria* exposure increased IL-33 release and promoted ILC2 and Th2 cell accumulation, with distinct temporal patterns between sexes. Cytokine and chemokine profiling revealed robust Th2, proinflammatory, and Th17 responses that varied with exposure time, with higher responses at ZT12 in females and ZT0 in males. Markers of tissue injury, including lactate dehydrogenase and uric acid, followed similar circadian-dependent trends. Functionally, *Alternaria* -exposed mice displayed greater AHR during the resting phase, particularly in males. Additionally, *Alternaria* disrupted epithelial junctional proteins (E-cadherin and ZO-1) and altered expression of core circadian clock proteins CLOCK, BMAL1, and REV-ERBα. Mechanistically, peak inflammation coincided with enhanced STAT1, STAT3, and STAT6 activation. Collectively, these findings identify circadian timing and sex as key determinants of fungal allergen-induced airway inflammation and suggest chronotherapeutic approaches may improve asthma outcomes.

## Full Text Availability

The license terms selected by the author(s) for this preprint version do not permit archiving in PMC. The full text is available from the preprint server.

